# Does ADT Influence the Risk of Suicidal Ideation among US Veteran Prostate Cancer Patients Pre-Exposed to PTSD?

**DOI:** 10.3390/cancers15102739

**Published:** 2023-05-12

**Authors:** Smit Brahmbhatt, Herta H. Chao, Shiv Verma, Sanjay Gupta

**Affiliations:** 1 College of Arts and Sciences, Case Western Reserve University, Cleveland, OH 44016, USA; ssb120@case.edu; 2 Department of Medicine & Yale Comprehensive Cancer Center, Yale University School of Medicine, New Haven, CT 06510, USA; herta.chao@yale.edu; 3 Yale Comprehensive Cancer Center & VA Connecticut Healthcare System, West Haven, CT 06516, USA; 4 Department of Urology, School of Medicine, Case Western Reserve University, Cleveland, OH 44106, USA; sxv304@case.edu; 5 The Urology Institute, University Hospitals Cleveland Medical Center, Cleveland, OH 44106, USA; 6 Department of Pharmacology, Case Western Reserve University, Cleveland, OH 44016, USA; 7 Department of Pathology, Case Western Reserve University, Cleveland, OH 44016, USA; 8 Department of Nutrition, Case Western Reserve University, Cleveland, OH 44016, USA; 9 Division of General Medical Sciences, Case Comprehensive Cancer Center, Cleveland, OH 44106, USA

## 1. Introduction

Post-traumatic stress disorder (PTSD) is defined as a mental health disease that has a high probability of developing among individuals who have experienced traumatic events. US veterans suffering from PTSD have experienced an increase in suicidal mortality rate [1]. US veterans pre-exposed to PTSD and diagnosed with other comorbidities, such as cancer, and its treatment course, such as androgen deprivation therapy (ADT) might influence severe cognitive disorders such as depression, anxiety, bipolar disorder, etc. [2,3]. This editorial aims to explore the possible association between ADT and the increased risk of suicidal ideation in US veteran males with pre-existing PTSD using the published scientific literature [1,4]. “We hypothesize that US veterans who have been pre-exposed to PTSD and are treated with ADT for prostate cancer will have higher rates of suicidal ideation.”

A study by Cooper et al. [1] determined whether US veterans who suffered from PTSD would have a higher risk of suicide mortality compared to US veterans who did not suffer from PTSD. They administered 1,693,449 primary care–post-traumatic stress disorder (PC-PTSD) screens to a total number of 1,552,581 US veterans all over the country. The PC-PTSD screening gathered information on patients in order to classify and diagnose them for PTSD. The screening utilized a score on a scale of 1–4. A score of 1–2 indicated a negative diagnosis of PC-PTSD, while a score of 3–4 indicated a positive diagnosis. It was found that all positive PC-PTSD (scores 3–4) showed a 58% increase in suicide mortality 1 day after screening, and a 26% increase in suicide mortality 1 year after screening. This is an incredible screening tool, which showed a strong association between PC-PTSD and suicide mortality. Moreover, a positive diagnosis of PC-PTSD with a score of 4 showed an association of 70% increase in suicide mortality 1 day after screening. No data were provided showing an increase in suicide mortality 1 year after screening. The study concluded that US veterans receiving a score of 4 on their PC-PTSD screening were associated with a significant increase in suicide mortality.

US veterans are exposed to several comorbidities, such as cancer [5], diabetes [6], cardiovascular disease [7], dementia [8], etc., which play a key role in influencing the onset of depression, linked to triggering suicidal ideation [2,9]. Dent et al. [10] have shown that US veterans pre-exposed to PTSD and diagnosed with lung, esophageal, or head and neck cancer were at elevated risk for suicidal ideation. Furthermore, the study did not find evidence for an increased suicidal risk in other cancer patients, including colorectal, pancreatic, prostate, bladder, gastric, and leukemia [10]. In another study, US veteran males with PTSD and diagnosis of prostate cancer experienced improved non-suicide survival despite being at greater suicide risk [11]. The most important therapeutic intervention for advanced-stage prostate cancer is ADT, which blocks the androgen synthesis in the body [12]. ADT remains the foundation of all treatments for men with advanced-stage metastatic prostate cancer even when they develop a need for additional therapies. Previous published studies have shown that long-term ADT exposure is linked with adverse cognitive dysfunction [13,14]. This dysregulation in brain health can cause effects such as mood changes [15], depression [16], dementia [17], and other cognitive impairments. ADT is shown to significantly increase the risk of depression and cognitive impairment [3,18]. Prostate cancer has been identified as a risk significant risk factor for late-life suicides in men within 6 months of diagnosis [19].

A study conducted in Lithuania by Patasius et al. [4] analyzed whether ADT treatment affects risk of suicide among prostate cancer patients. The study separated the dataset of 8908 men with advanced prostate cancer by ADT use (users/nonusers), age (65<, 65–74, 75+), and extent of their disease (Stage III and IV). Primary and secondary statistical analysis was used to determine if there was a significant correlation between ADT and increase in risk of suicide. The results showed that ADT is a possible risk factor for suicide in men who suffer from prostate cancer, compared to men suffering from prostate cancer who are nonusers of ADT. Men undergoing ADT treatment, in multivariate analysis after adjustment of age at diagnosis and stage, showed a higher risk of suicide mortality compared to the general population. It was also found that age and stage of cancer were important risk factors for suicide. Finally, the study concluded that even after age and stage of cancer were adjusted for, there was still an increased risk of suicide among prostate cancer patients who were on ADT. The above observation warrants further research and investigation of the potential link between ADT and the aggravation of PTSD symptoms.

## 2. Association between ADT and Suicidal Ideation

Considering the studies mentioned above [1,4], there may be an underlying link between ADT and US veterans with pre-existing PTSD, which may exacerbate the risk of suicidal ideation. PTSD alone has been linked to a higher risk of suicidal ideation and successful attempts [20]. Other studies have alluded to the possibility that ADT causes an increase in the risk of depression, and consequently an increase in suicidal thought [11]. Treatment of prostate cancer utilizing ADT, extreme reduction of androgen levels in males, does reduce the progression of cancer cells, but it can also have adverse outcomes [21], such as sexual dysfunction, fatigue, infertility, osteoporosis, and cardiovascular disease among others, which could play a key role in the triggering of suicidal ideation.

A study conducted by Aboumrad et al. [11] explored the risk of suicide and non-suicide mortalities among US veterans who were newly diagnosed with prostate cancer [11]. They found that US veteran patients diagnosed with PTSD experienced a higher rate of suicide mortality when compared to US veteran patients who were not diagnosed with PTSD. They identified depression, substance use disorder, and prostate cancer treatment as partial mediators. After adjustment for these mediators, it was found that US veterans were still at a higher risk of suicide.

Prostate cancer diagnosis has been found to exacerbate PTSD and increase suicidal risk among US veterans. The combination of both PTSD and potential side-effects of ADT could generate troubling mental health challenges, but the precise role and impact of ADT on PTSD and suicidality remains to be clarified and warrants further investigation. Based on the above, our hypothesis supports the continuous monitoring of US veterans who have been pre-exposed to PTSD and are treated for prostate cancer under surveillance with psycho-oncologists, mental health therapists, and psychiatrists. Additionally, new methods of treating prostate cancer among US veterans should be explored and evaluated.

## 3. Current Research and Limitations

Some of the conducted research studies help provide a better understanding as to whether ADT could cause PTSD symptoms to be expressed at elevated intensities. However, more research is required to provide a definitive link. Most ADT and PTSD studies conducted have been directed toward the general population and not the US veteran population. Another factor that proves to be a limitation is that it is extremely hard to determine control groups, and most data related to suicide are self-reported (suicidal thoughts and ideations), which poses an obstacle to the validity of the study. There may also be potential sources of bias or confusion among patients. It is necessary for a large-scale study to be conducted with a reliable control group in order to understand the true effects of ADT on elevating suicidal ideation caused by PTSD. Scientists should explore the use of FLAG-tag blood-based biomarkers either at the gene or protein level, which might distinguish the severity of mental illness and allow for early detection and prevention.

Other limitations that researchers may face are the confounding factors which can directly and indirectly influence the symptoms of PTSD, such as the socioeconomic situation of the US veteran and their family, governmental assistance, the present environment that they live and work in, separation from family members, etc.

## 4. Conclusions

It is evident that among US veterans, there is a significant level of PTSD, as well as a considerable number of prostate cancer patients being treated with ADT. Finding the effects of combining the two can help healthcare professionals provide better care to US veterans. There is an urgent need for researchers to further explore this area of interest. This can be achieved by finding key biomarkers for patients suffering with PTSD and prostate cancer which tag patients with a need for mental health therapists and psychiatrists.

Meanwhile, healthcare professionals should critically consider the risks of ADT on the mental health of US veterans suffering from PTSD. It is of prime importance that close medical supervision should be provided throughout the treatment. Adequate mental health support should be integrated into the treatment plan to ensure that the mental health aspect of the patient is carefully controlled.
